# Macular Bruch´s Membrane Length and Axial Length. The Beijing Eye Study

**DOI:** 10.1371/journal.pone.0136833

**Published:** 2015-08-28

**Authors:** Jost B. Jonas, Ya Xing Wang, Qi Zhang, Yi Liu, Liang Xu, Wen Bin Wei

**Affiliations:** 1 Beijing Institute of Ophthalmology, Beijing Tongren Eye Center, Beijing Tongren Hospital, Capital Medical University, Beijing Ophthalmology and Visual Science Key Lab, Beijing, China; 2 Department of Ophthalmology, Medical Faculty Mannheim of the Ruprecht-Karls-University of Heidelberg, Germany; 3 Beijing Tongren Eye Center, Beijing Tongren Hospital, Capital Medical University, Beijing, China; Medical College of Soochow University, CHINA

## Abstract

**Purpose:**

To assess whether macular Bruch´s membrane gets lengthened in axial myopia.

**Methods:**

Using the enhanced depth imaging mode of spectral-domain optical coherence tomography and examining a subgroup of participants of the population-based cross-sectional Beijing Eye Study, we measured the length of Bruch´s membrane (“MacBMLength”) from the fovea to the temporal edge of parapapillary gamma zone, and the distance between the fovea and the temporal optic disc border. Parapapillary gamma zone was defined as the parapapillary region without Bruch´s membrane. We additionally measured ocular biometric parameters and assessed non-ophthalmologic variables.

**Results:**

Measurements of MacBMLength were performed on 322 individuals. MacBMLength (mean: 3.99±0.33 mm; range: 3.17–4.93 mm) was not significantly associated with any systemic parameter or ocular biometric parameter. Gamma zone width (mean: 0.18±0.30mm; range: 0.00–2.61mm) was associated (multivariate analysis; correlation coefficient r:0.80) with longer axial length (*P*<0.001; standardized correlation coefficient beta: 0.60; non-standardized correlation coefficient B:0.11; 95%CI: 0.09,0.14) and with longer fovea-optic disc border distance (*P*<0.001; beta:0.28; B:0.19; 95%CI:0.14,0.25), but not with MacBMLength (*P* = 0.42). Fovea-temporal disc border distance (mean: 4.16±0.44mm; range: 3.17–5.86mm) was associated (overall correlation coefficient: 0.68) with longer axial length (*P*<0.001; beta: 0.36; B: 0.10; 95%CI: 0.06, 0.13), after adjusting for flatter anterior chamber depth (*P* = 0.003; beta:-0.14; B:-0.14; 95%CI: -0.23,-0.05) and wider parapapillary gamma zone (*P*<0.001; beta:0.42; B:0.62; 95%CI:0.44,0.81).

**Conclusions:**

In contrast to parapapillary gamma zone width and fovea-disc border distance, MacBMLength was not significantly associated with axial length. Axial elongation associated increase in fovea-disc distance may predominantly occur through development or elongation of parapapillary gamma zone, while macular Bruch´s membrane may mostly be independent of axial elongation.

## Introduction

Axial myopization is associated with an elongation of the globe diameters, more in the sagittal direction than in the horizontal direction or the vertical direction. It leads to a change in the shape of the eye, from a mostly spherical shape in the case of emmetropic globes to an axially elongated shape in highly myopic eyes. Based upon measurements of choroidal and scleral thickness, histomorphometric studies have revealed that the axial elongation associated thinning of the two outer ocular layers is found mostly posterior to the equator and that it is more marked the closer to the posterior pole [1–4]. If the axial elongation associated changes occur predominantly at the posterior pole, one may infer that the size of the macula increases. If the macular region enlarges postnatally and assuming that the retinal photoreceptors do not multiply after birth or after the end of the second year of life, one may conclude that the density of the retinal photoreceptors in the macular region decreases and that the inter-photoreceptor distance subsequently increases with longer axial length. This would imply a decreased visual acuity in axially myopic eyes versus in emmetropic eyes. It would be contradictory to the results of previous population-based studies in which, after exclusion of highly myopic eyes, best corrected visual acuity was not significantly associated with axial length in a multivariate analysis [5]. Since the retinal photoreceptors rest on the retinal pigment epithelium which forms Bruch´s membrane as its basal membrane, the density of the foveal photoreceptors will depend on a potential lengthening of macular Bruch´s membrane in axially myopic eyes.

In particular in some myopic eyes, Bruch´s membrane does not reach the temporal optic disc border but leaves a parapapillary temporal region free of Bruch´s membrane [6–16]. That region has recently been called “parapapillary gamma zone” [14–16]. We therefore conducted the current study to investigate the association between the distance between the fovea and the end of Bruch´s membrane in direction to the optic disc (macular Bruch´s membrane length or “MacBMLength”), parapapillary gamma zone, the fovea-disc border distance, and axial length. The width of parapapillary gamma zone was calculated as difference of fovea-disc border distance minus MacBMLength.

## Methods

The Beijing Eye Study 2011 is a population-based investigation performed in a rural and an urban region of Beijing. The study protocol was approved by the Medical Ethics Committee of the Beijing Tongren Hospital and informed written consent was obtained from all study participants. Out of 4403 eligible individuals fulfilling the inclusion criterion of an age of 50+ years, 3468 (78.8%) individuals participated. The mean age was 64.6 ± 9.8 years (median, 64 years; range, 50–93 years). The study has been described in detail previously [17,18]. Exclusion criterion for the present study was the presence of macular defects in Bruch´s membrane as described in detail previously [19,20]. Additional inclusion criterion for the present investigation was the availability of measurements of the MacBMLength and of the fovea–temporal optic disc border distance.

**Fig 1:**
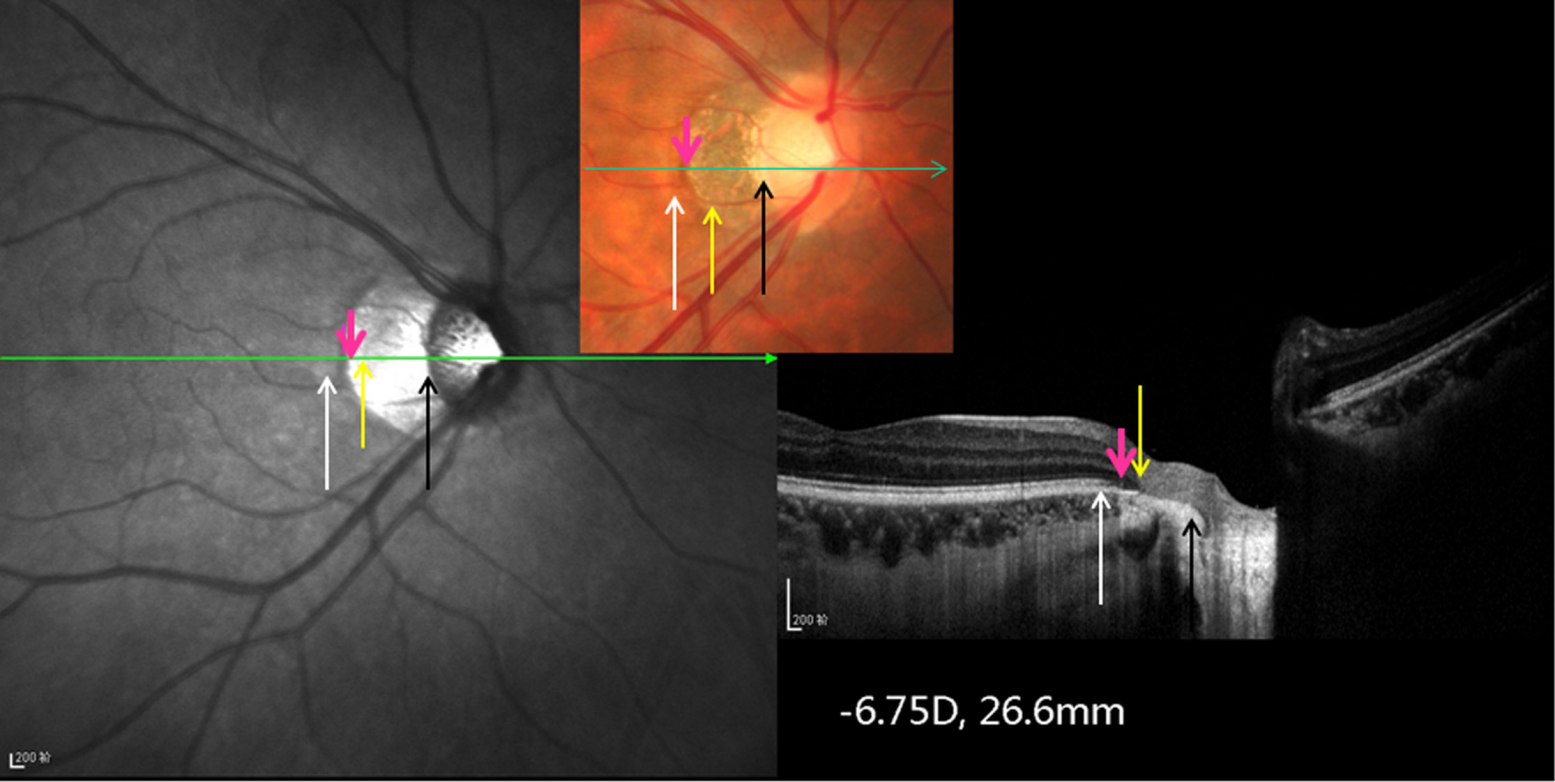
Optical coherent tomographic image and corresponding optic disc photograph of an eye with parapapillary gamma zone. White arrow to pink arrow: parapapillary alpha zone with irregular retinal pigment epithelium; pink arrow to yellow arrow: parapapillary beta zone with presence of Bruch´s membrane and absence of retinal pigment epithelium; yellow arrow to black arrow: parapapillary gamma zone with absence of Bruch´s membrane; black arrow: optic disc border

**Fig 2:**
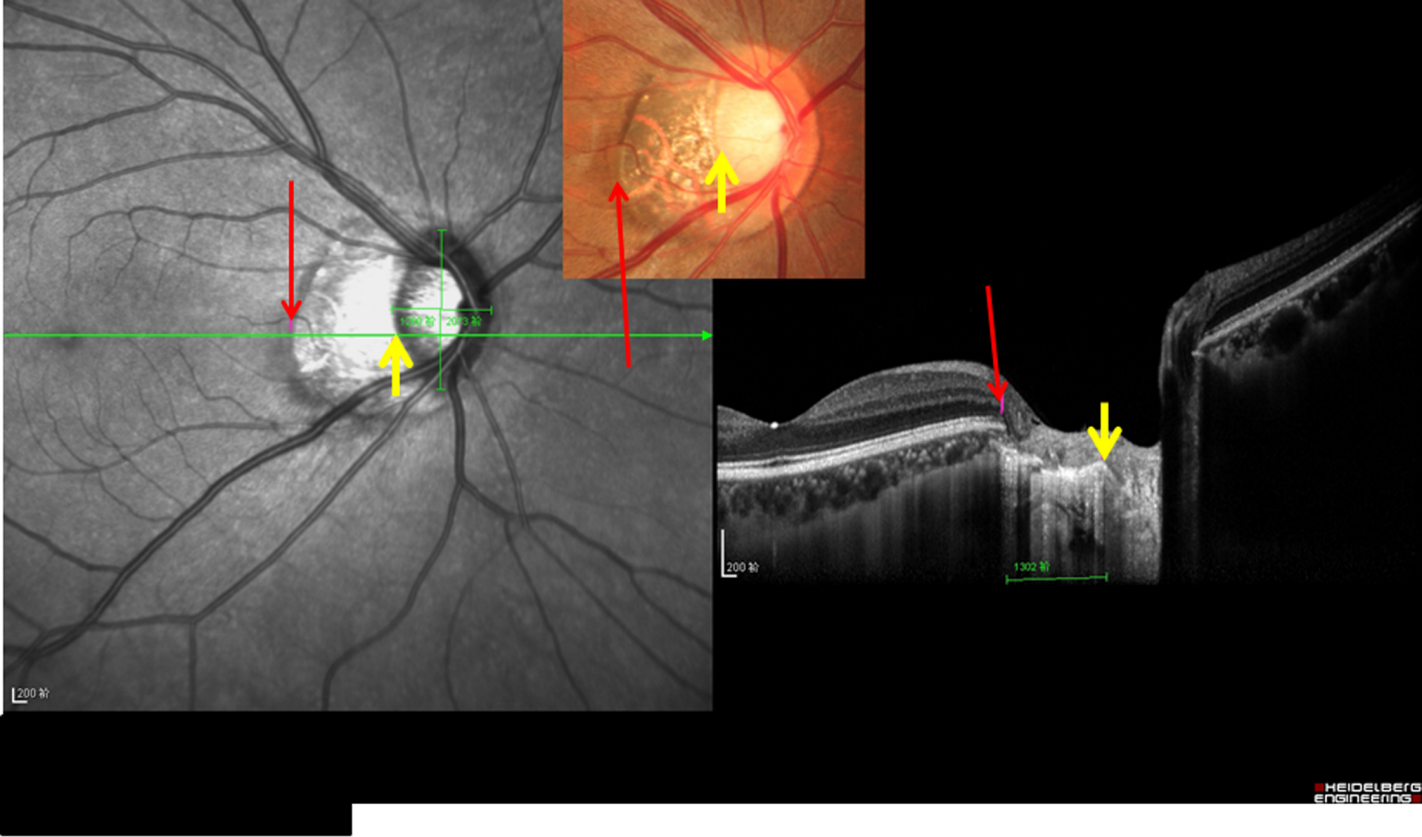
Optical coherent tomographic image and corresponding optic disc photograph of an eye with parapapillary gamma zone. Long red arrow: end of Bruch´s membrane; yellow arrow: optic disc border

All participants underwent an interview with a structured questionnaire, biochemical blood examinations, measurement of blood pressure and body height and weight, and a comprehensive ophthalmic examination. We measured visual acuity and performed a slit lamp examination of the anterior and posterior segment of the eye and a digital photography of the cornea, lens, macula and optic disc (fundus camera Type CR6-45NM; Canon Inc., Tokyo, Japan). Spectral domain optical coherence tomography (SD-OCT, Spectralis, Heidelberg Engineering Co., Heidelberg, Germany) with enhanced depth imaging modality was carried out after pupil dilation [21]. Thirty-one OCT sections were obtained, which covered a 30° x 30° large rectangle centered around the fovea. Using the enhanced depth imaging mode, seven additional sections (each comprising 100 averaged scans) were obtained in a 5° x 30° large rectangle centered onto the fovea. Using the scan running through the fovea (which was identified as the point of the maximal foveal depression), we measured the length of Bruch´s membrane from the fovea to the end of Bruch´s membrane in direction of the optic nerve head. Using the fundus photographs, we measured the distance between the fovea and the temporal border of the optic nerve head. In the parapapillary region, we differentiated between an alpha zone characterized by the presence of Bruch´s membrane with irregular retinal pigment epithelium, beta zone characterized by the presence of Bruch´s membrane and absence of retinal pigment epithelium, and gamma zone characterized by the absence of Bruch´s membrane (Figs 1 and 2). We corrected the image magnification caused by the optic media of the eye and by the fundus camera using the Littmann-Bennett method [22–24].

Statistical analysis was performed using a commercially available statistical software package (SPSS for Windows, version 22.0, IBM-SPSS, Chicago, IL, USA). The study population was divided in subgroups by axial length, and within each subgroup, we randomly selected participants to be included into the present study. We examined the distribution of the MacBMLength and of the fovea-disc border distance using the Kolmogorov-Smirnov test and determined the mean ± standard deviations of the parameters. We calculated the length of the parapapillary region without Bruch´s membrane (gamma zone) as the difference of the fovea-disc border distance minus the MacBMLength. We then performed a univariate analysis of the associations between the MacBMLength, the fovea-disc border distance or the width of gamma zone with other ocular and systemic variables. Finally, we conducted a multivariate regression analysis, with the MacBMLength, the fovea-disc border distance or gamma zone width as the dependent variable and all those parameters as independent variables which were significantly associated with dependent parameter in the univariate analysis. From the list of independent parameters we then dropped step by step those parameters which were no longer significantly associated. 95% confidence intervals (CI) were presented. All *P*-values were two-sided and were considered statistically significant if the values were smaller than 0.01.

## Results

Out of the 3468 subjects, measurements of MacBMLength and of the fovea–temporal optic disc border distance were performed for 322 individuals (173 women). Mean age was 62.9 ± 8.8 years (median: 61 years; range: 50–90 years), mean axial length was 24.2 ± 1.7 mm (median: 23.9 mm; range: 21.2 to 30.9 mm), and mean refractive error was -3.08 ± 3.06 diopters (median: -2.50 diopters; range: -20.00 to +2.00 diopters). Due to the selection of the study participants, the group of subjects with measurements of MacBMLength and of the fovea- temporal disc border distance as compared with the group of individuals without these measurements was significantly (*P*<0.001) younger (62.8 ± 8.8 years versus 64.8 ± 9.9 years), had a significantly (*P*<0.001) longer axial length (24.3 ± 1.7 mm versus 23.1 ± 1.0 mm) and was significantly (*P*<0.001) more myopic (-3.08 ± 3.04 diopters versus 0.09 ± 1.72 diopters), while both groups did not differ significantly in gender (*P* = 0.34).

Mean fovea–disc border distance was 4.16 ± 0.44 mm (median: 4.07 mm; range: 3.17–5.86 mm), and mean MacBMLength was 3.99 ± 0.33 mm (median: 3.98 mm; range: 3.17 to 4.93 mm). The width of gamma zone correspondingly measured 0.18 ± 0.30 mm (median: 0.04 mm; range: 0.00–2.61 mm). MacBMLength and the fovea–disc border distance were both normally distributed (*P* = 0.20), while width of gamma zone was not (*P*<0.001).

**Fig 3:**
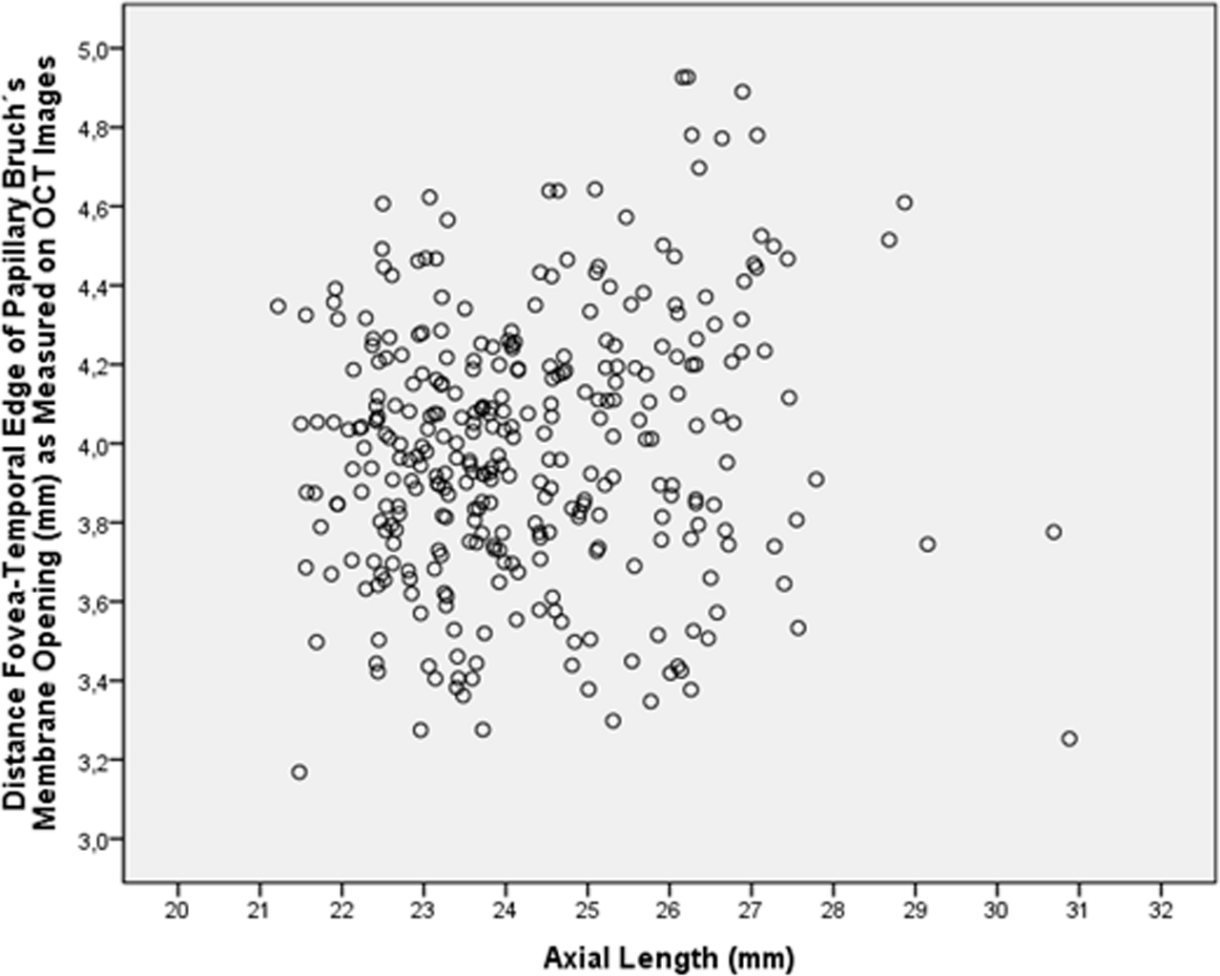
Scattergram showing the distribution of the macular Bruch´s membrane length (distance between the fovea and the temporal edge of the papillary Bruch´s membrane opening) in relationship to axial length.

In univariate analysis, MacBMLength was marginally associated only with longer axial length (*P* = 0.02; correlation coefficient r: 0.13) (Fig 3). The MacBMLength was not significantly associated with age (*P* = 0.10), gender (*P* = 0.46), higher body height (*P* = 0.10), body mass index (*P* = 0.20), systolic blood pressure (*P* = 0.45), diastolic blood pressure (*P* = 0.14), blood concentrations of glucose (*P* = 0.41), high-density lipoproteins (*P* = 0.45), cholesterol (*P* = 0.21), low-density lipoproteins (*P* = 0.23), and triglycerides (P = 0.93), HbA1c value (*P* = 0.08), thicker central corneal thickness (*P* = 0.10), anterior chamber depth (*P* = 0.20), anterior corneal curvature radius (*P* = 0.98), lens thickness (*P* = 0.27), intraocular pressure (*P* = 0.89), optic disc-fovea angle (*P* = 0.39), optic disc area (*P* = 0.79), prevalence of early age-related macular degeneration (*P* = 0.81), intermediate age-related macular degeneration (*P* = 0.73) and of any age-related macular degeneration (*P* = 0.98), prevalence of cortical cataract (*P* = 0.78), nuclear cataract (*P* = 0.52) and subcapsular cataract (*P* = 0.87), prevalence of open-angle glaucoma (*P* = 0.60), angle-closure glaucoma (*P* = 0.98), diabetic retinopathy (*P* = 0.07; r: 0.10) and retinal vein occlusions (*P* = 0.46; r: -0.04).

**Fig 4:**
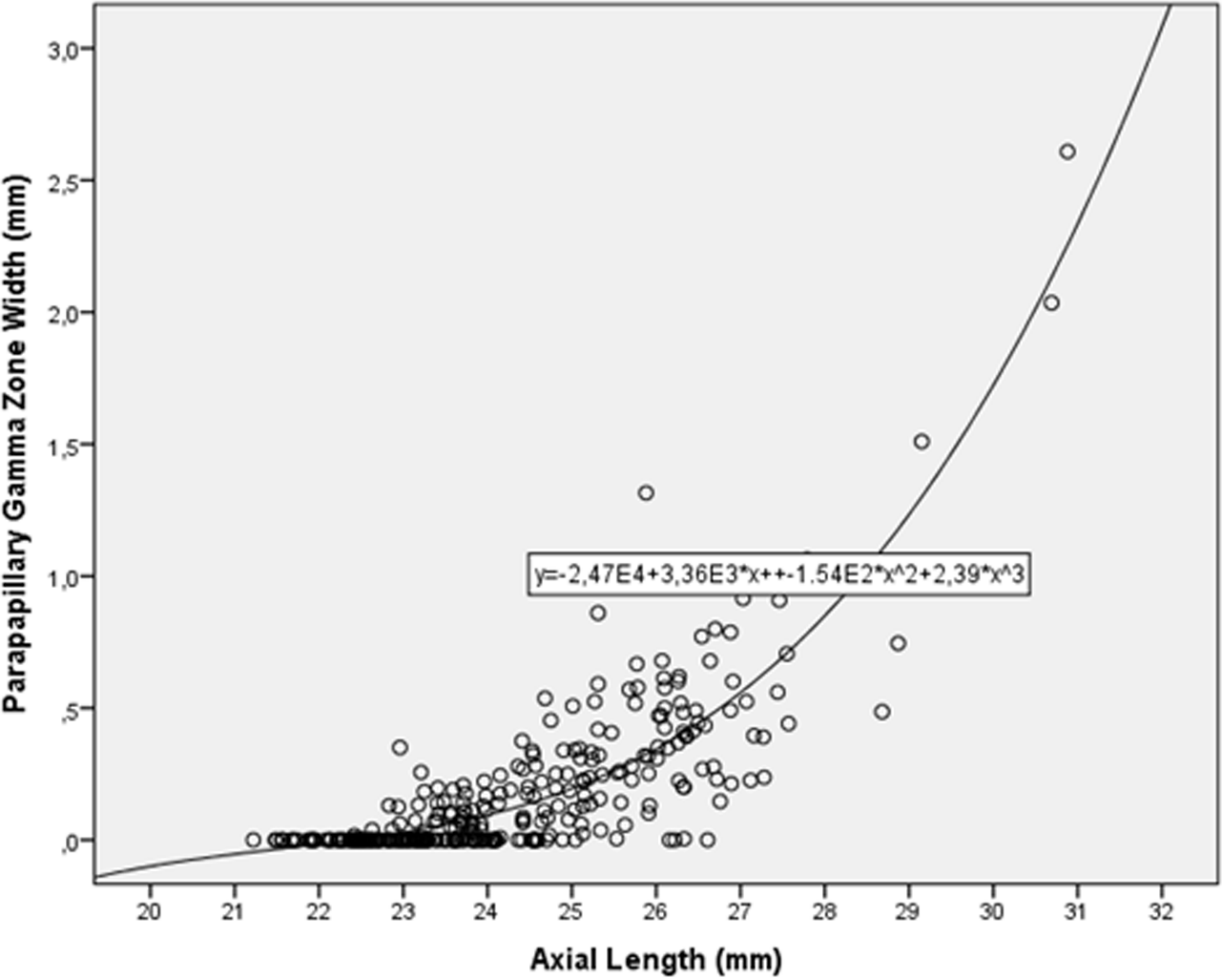
Scattergram showing the distribution of the width of parapapillary gamma zone in relationship to axial length (Correlation Coefficient r^2^: 0.73)

**Fig 5:**
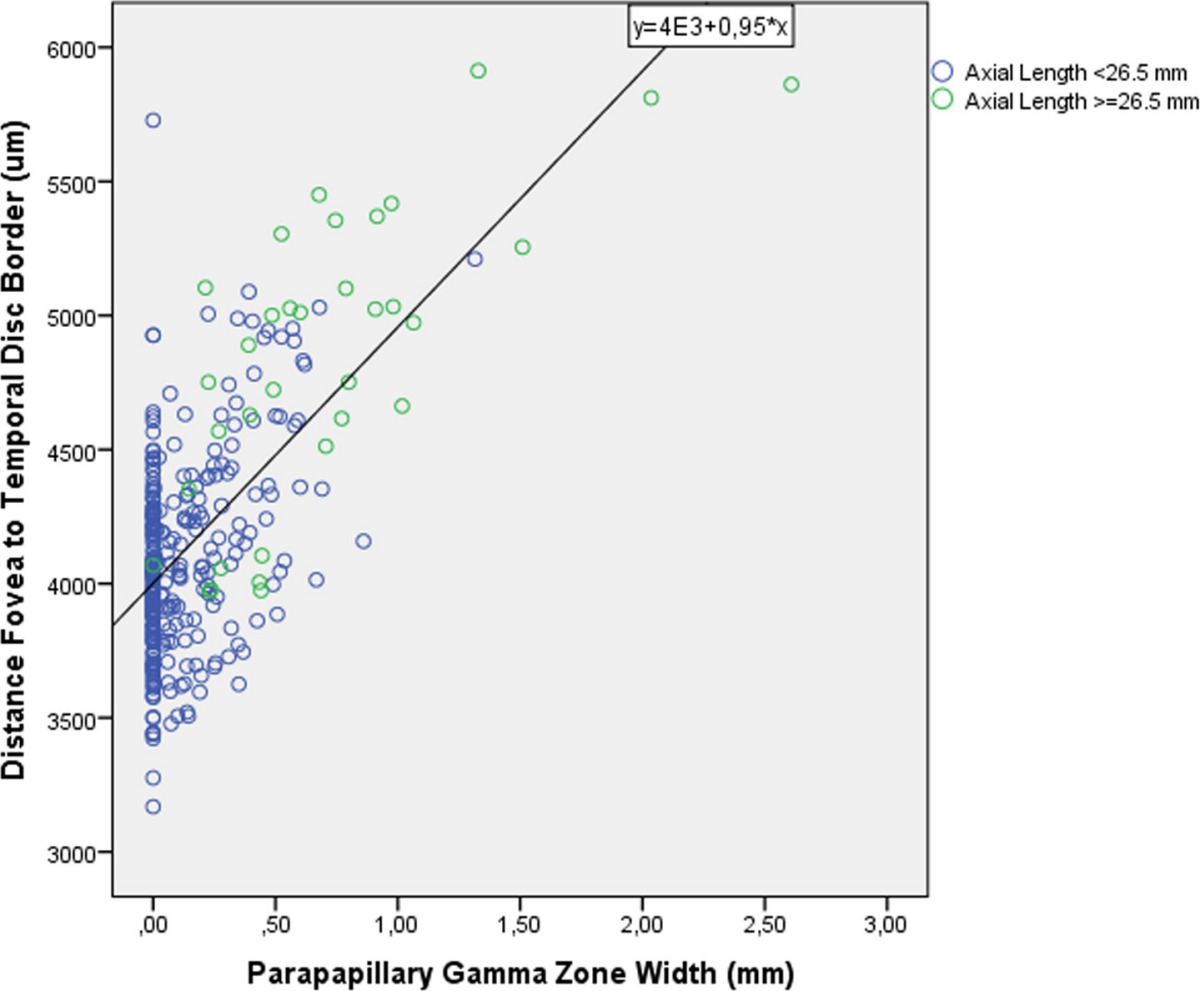
Scattergram showing the distribution of the width of parapapillary gamma zone in relationship to the fovea-disc border distance

**Fig 6:**
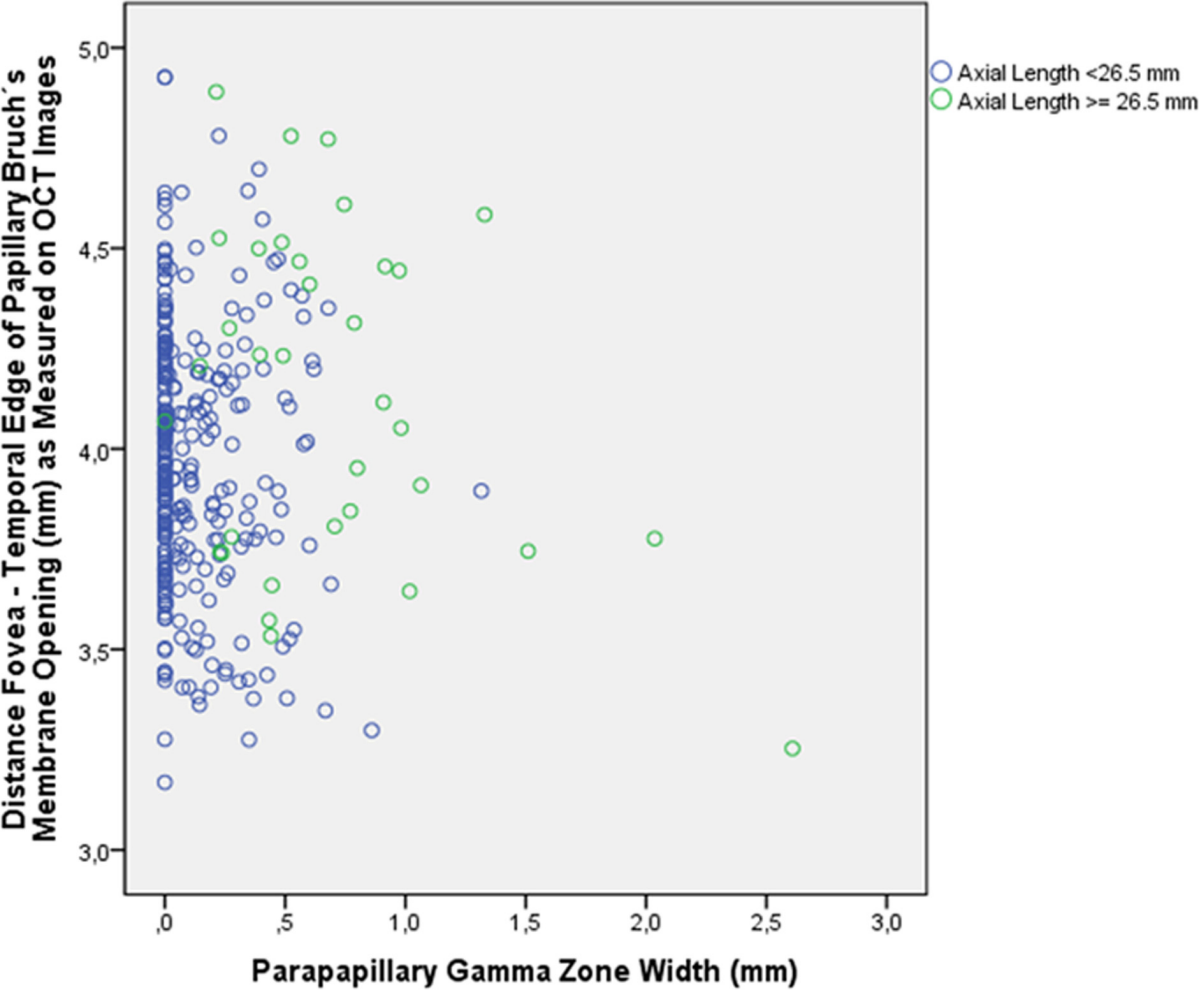
Scattergram showing the relationship between the width of parapapillary gamma zone in relationship to macular Bruch´s membrane length (distance between the fovea and the temporal edge of the papillary Bruch´s membrane opening)

Gamma zone width was significantly (univariate analysis) associated with axial length (*P*<0.001; r: 0.76) (Fig 4) and with the fovea-optic disc border distance (*P*<0.001; r: 0.65) (Fig 5), while it was not significantly associated with MacBMLength (*P* = 0.42) (Fig 6). In multivariate analysis, larger gamma zone width was associated (correlation coefficient r: 0.80) with longer axial length (*P*<0.001; standardized correlation coefficient beta: 0.60; non-standardized correlation coefficient B: 0.11; 95%CI: 0.09, 0.14) and with longer fovea-optic disc border distance (*P*<0.001; beta: 0.28; B: 0.19; 95%CI: 0.14, 0.25).

**Fig 7:**
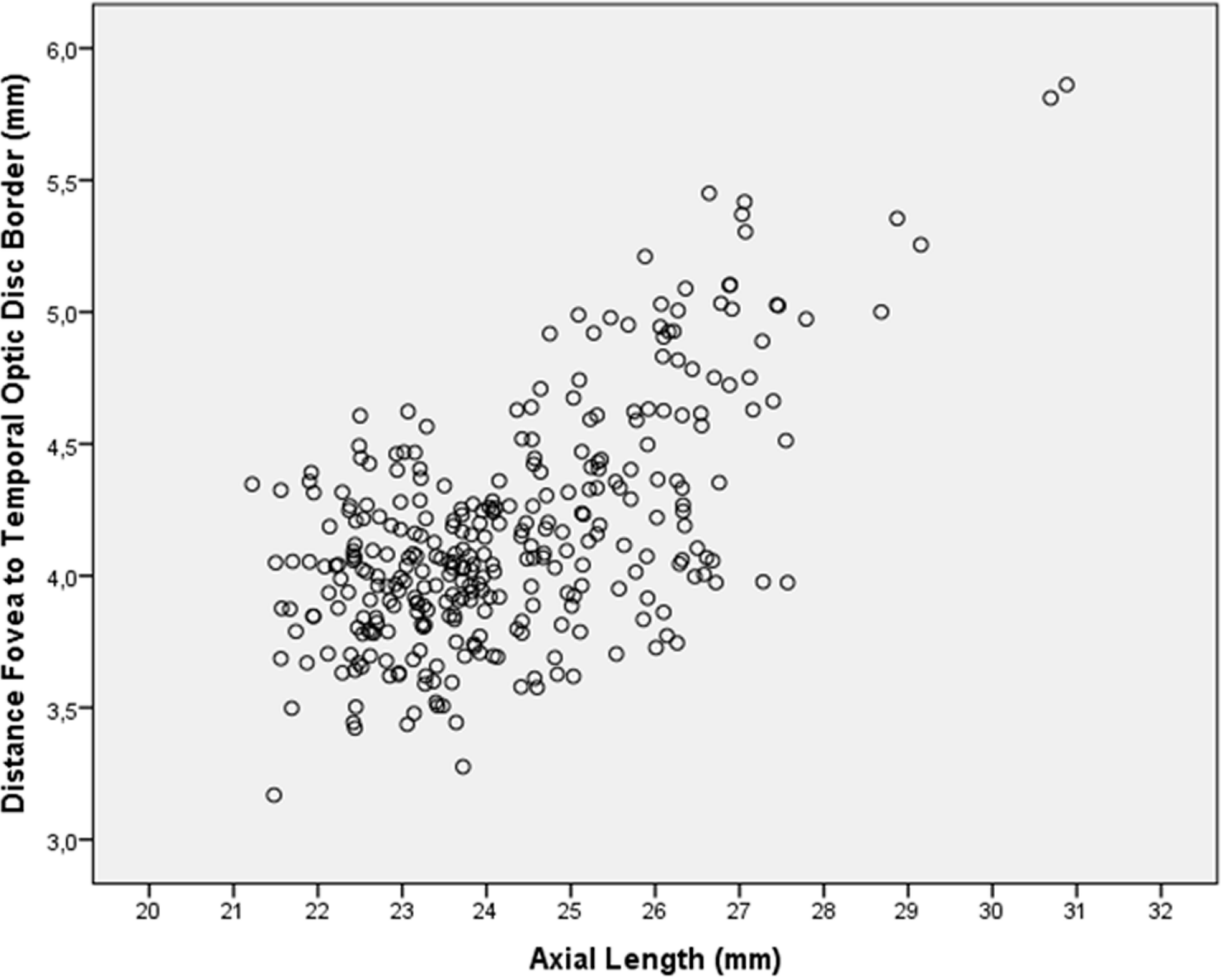
Scattergram showing the relationship between the distance between fovea and the temporal optic disc border as compared with axial length

Mean fovea-optic disc border distance was 4.16 ± 0.44 mm (median: 4.07 mm; range: 3.17–5.86 mm). In univariate analysis, fovea-optic disc border distance was significantly associated with the systemic parameters of urban region of habitation (*P*<0.001, correlation coefficient r: 0.35), higher level of education (*P*<0.001; r: 0.29), and with the ocular parameters of longer axial length (*P*<0.001; r: 0.62) (Fig 7), deeper anterior chamber depth (*P*<0.001; r: 0.20), larger anterior corneal curvature radius (*P*<0.001; r: 0.20), and wider parapapillary gamma zone (*P*<0.001; r: 0.65). The fovea-optic disc border distance was not significantly associated with age (*P* = 0.09; r: 0.09), gender (*P* = 0.81), body mass index (*P* = 0.14), body height (*P* = 0.18; r: 0.08), body weight (*P* = 0.67), diastolic blood pressure (*P* = 0.02; r: -0.14), systolic blood pressure (*P* = 0.02; r: -0.14), blood concentration of glucose (*P* = 0.81), value of HbA1c (*P* = 0.75), blood concentration of low-density lipoproteins (*P* = 0.08; r: 0.11), high-density lipoproteins (*P* = 0.13, r: -0.19), cholesterol (*P* = 0.08; r: 0.11), and triglycerides (*P* = 0.90), central corneal thickness (*P* = 0.02; r: 0.13), thinner lens thickness (*P*<0.90), disc-fovea angle (*P* = 0.36), and optic disc area (*P* = 0.80).

In multivariate analysis, longer fovea-optic disc border distance remained to be significantly associated (overall correlation coefficient: 0.68) with longer axial length (*P*<0.001; beta: 0.36; B: 0.10; 95%CI: 0.06, 0.13), flatter anterior chamber depth (*P* = 0.003; beta: -0.14; B: -0.14; 95%CI: -0.23, -0.05) and wider parapapillary gamma zone (*P*<0.001; beta: 0.42; B: 0.62; 95%CI: 0.44, 0.81).

## Discussion

MacBMLength was not significantly associated with any systemic parameter or ocular biometric parameter except for, if at all, a weak relationship with longer axial length (*P* = 0.02; r^2^: 0.02) (Fig 3). The value of the correlation coefficient indicated that 2% of the variation in MacBMLength could be explained by a variation in axial length. Parapapillary gamma zone width was strongly associated with longer axial length (*P*<0.001; r:0.60) but not with MacBMLength (*P* = 0.42). The fovea-temporal disc border distance was strongly associated with longer axial length (*P*<0.001; beta: 0.36) after adjusting flatter anterior chamber depth (*P* = 0.003; beta: -0.14) and wider parapapillary gamma zone (*P*<0.001; beta: 0.42). The results suggest that the axial elongation associated increase in the fovea-disc border distance predominantly occurred through a development or elongation of parapapillary gamma zone, while macular Bruch´s membrane was mostly independent of axial elongation. One may infer that in axial elongation with a mostly constant macular Bruch´s membrane length, the density of retinal photoreceptors in the fovea remained unchanged.

The result of our study on an increase in the fovea–optic disc distance agrees with previous investigations [9,25]. It may be explained by the finding that myopic axial elongation leads to changes which are more marked the closer to the posterior pole [1–4]. Histomorphometric investigations revealed that changes associated with primary high axial myopia are markedly less detectable anterior to the equator [1–4]. It is in contrast to eyes with secondary high axial myopia due to congenital glaucoma, in which scleral thinning occurs posterior and anterior to the equator and also includes a widening and thinning of the cornea.

The width of gamma zone as assessed in our study (0.18 ± 0.30 mm) roughly corresponded to the mean distance from the temporal disc margin to the edge of Bruch´s membrane as measured in the study by Park and colleagues (0.21 ± 0.20 mm) [6].

The finding of our investigation on an axial length dependent increase in the width of parapapillary gamma zone is in agreement with previous histomorphometric studies and clinical studies [8,9,14–16]. In these studies, one had differentiated between alpha zone, characterized by the presence of Bruch´s membrane and presence of an irregularly structured retinal pigment epithelium; beta zone defined by the presence of Bruch´s membrane and absence of retinal pigment epithelium and which was located between the peripheral alpha zone and the optic disc border, or between alpha zone and gamma zone if gamma zone was present; and gamma zone, defined by the absence of Bruch´s membrane and located directly at the peripapillary ring which forms the border optic nerve head [14,26]. In the preceding studies as well as in the present investigation, gamma zone strongly increased with longer axial length, while it was not, or only marginally, associated with glaucoma. In contrast, parapapillary beta zone was significantly associated with glaucoma, while it was not, or not markedly, associated with axial length [14–16]. Also in our study, gamma zone increased with longer axial length, with the association starting mostly beyond an axial length of 25 mm (Fig 4). These results agree with findings obtained in other previous investigations. On the images of 72 healthy adults, Chui and colleagues measured the fovea–temporal optic disc border distance to be 4.22 mm ± 0.46 and the distance between the foveola and the temporal edge of the peripapillary crescent to be 3.97 mm ± 0.25 mm [9]. The difference between both measurements (approximately corresponding to parapapillary gamma zone in pour study) was similar to the width of gamma zone as determined in our study. In Chui´s study (as in our study), only the fovea–temporal optic disc border distance was significantly correlated with axial length. As axial length increased by 10% in Chui´s investigation, the fovea–temporal optic disc border distance increased by 13%, while the outer neural retina only expanded by 4%. Chui and colleagues concluded that retinal stretching might not mirror scleral growth. Nonaka and coworkers examined 61 highly myopic (≥ -6 diopters) eyes without myopic retinopathy [8]. They found that the distance from the fovea nasal margin of a socalled PPA-ß zone (presumable gamma zone) correlated with axial length (*P*<0.05; correlation coefficient r: 0.26), and that the width of the PPA-ß zone correlated with axial length (*P*<0.05; r: 0.32).

The parapapillary gamma zone as it was called in our study has previously been addressed in previous investigations [6–16,27–31]. Applying Fourier-domain optical coherence tomography, Park and coworkers found that within a clinical parapapillary beta zone atrophy the end of Bruch´s membrane did not reach the border of the optic nerve head in all eyes [6]. This zone without Bruch´s membrane would be the equivalent of gamma zone in our study. Hayashi and associates reported that a parapapillary beta zone as defined by these authors either included a Bruch's membrane which was straight or downward-curved or which showed a downward-bending slope without Bruch´s membrane [29]. This latter region without Bruch´s membrane was called “gamma” zone in our investigation. Lee and coworkers reported similar observations [7].

In contrast to the width of parapapillary gamma zone and to the total fovea-disc border distance, the length of macular Bruch´s membrane, defined as the distance between the fovea and the temporal edge of parapapillary gamma zone was mostly independent of axial length (Fig 3). It suggests that the axial elongation associated increase in the fovea-disc border distance led to the appearance or enlargement of a papillary gamma zone. This finding makes one infer that the distance between the retinal photoreceptors in the macular and foveal region was not markedly dependent on axial length as long as highly myopic eyes with secondary macular Bruch´s membrane defects were excluded [15,18]. Correspondingly, a recent multivariate analysis revealed that within non-highly myopic eyes (i.e. eyes with an axial length of less than 26 mm), better best corrected visual acuity was significantly associated with thicker subfoveal choroid (*P*<0.001) in general and a subfoveal choroid thicker than 30 μm (*P*<0.001) in particular, while it was not significantly with axial length, after adjusting for younger age (*P*<0.001), higher level of education (*P*<0.001), taller body stature (*P*<0.001), higher body mass index (*P* = 0.005), and absence of major ocular diseases such as glaucoma [5]. That finding also agrees with the results of the study by Li and associates who used an adaptive optics scanning laser ophthalmoscopy under optimized wavefront correction and measured the cone photoreceptor diameter in 18 healthy eyes with axial lengths ranging between 22.86 to 28.31 mm [32]. Ocular biometry and an eye model were used to estimate the retinal magnification factor. They found a significant decrease in cone density (*P*<0.05) with increasing axial length at an eccentricity of 0.30 or more mm, but not closer to the fovea. These results were contradicted the findings obtained in the investigation by Kitaguchi and colleagues who applied an adaptive optics fundus camera and examined 19 healthy individuals with a mean axial length ranging between 23.4 and 28.0 mm. They found that the average cone spacing in the moderate- to high-myopia group (4.71 ± 0.44 μm) was significantly greater (*P* = 0.002) than in the normal and low-myopia groups (3.90 ± 0.47 μm). The cone spacing was significantly correlated with the axial length (r: 0.77; *P*<0.001) [33]. Also, Chui reported that cone photoreceptor packing density (cells per square millimeter) was significantly lower in myopic eyes than in emmetropic eyes [34]. At a given location, there was considerable individual variation in cone photoreceptor packing density, although more than 20% of the variance could be accounted for by differences in axial length.

The finding that parapapillary gamma zone width was, but that macular Bruch´s membrane length was not, significantly associated with longer axial length showed that the axial elongation associated increase in the fovea-disc border distance was predominantly due to the development or widening of parapapillary gamma zone. As shown in Fig 5, the steepness of the regression line of the association between the fovea-disc border distance and the width of parapapillary gamma zone was 0.95, indicating that 95% of the increase in fovea-disc border distance was due to the increase in the gamma zone width. This can be regarded as the widening of the physiological defect in Bruch´s membrane. This primary Bruch´s membrane defect forms the inner layer of the optic nerve head. The latter can be considered to be a three-layered hole, with Bruch´s membrane hole or opening as the inner layer, the choroidal hole or defect as the middle layer, and the scleral hole as the outer hole. One may speculate that in the early years of life, all three holes are aligned to each other. If axial elongation occurs, the papillary Bruch´s membrane opening may move in direction to the foveal center while the scleral hole may stay behind, leading to an overhanging of Bruch´s membrane on the nasal disc side and a corresponding lack of Bruch´s membrane on the temporal side of the optic nerve head [35]. The lack of Bruch´s membrane on the temporal side of the optic nerve head is equivalent to the development of parapapillary gamma zone. The peripapillary ring is the continuation of the optic nerve pia mater and marks the peripheral border of the lamina cribrosa within the optic nerve head [26]. In agreement with the finding that the length of macular Bruch´s membrane did not enlarge with longer axial length agrees with a recent histomorphometric study that the thickness of Bruch´s membrane, in contrast to a scleral and choroidal thickness, did not decrease with longer axial length [36]. It suggests that the myopic axial elongation was not associated with a stretching and lengthening of Bruch´s membrane in the macular region, at least not in the region extending from the fovea into nasal direction. The question then arises, why some eye with high axial myopia can develop secondary Bruch´s membrane defects in the macular regions as described recently [15,20].

The constancy of macular Bruch´s membrane length and the increase in the fovea-disc border distance by an enlargement of parapapillary gamma zone may imply that Bruch´s membrane is not strongly fixed on the underlying sclera through the choroid. Such a theory of a sliding Bruch´s membrane has also been discussed in a previous study in which the parapapillary region, presumably gamma zone, showed a marked decrease in size after a profound reduction in intraocular pressure for several months had occurred [37]. The concept of a Bruch´s membrane sliding by the swamping choroid on the sclera does not contradict a firm and stable relationship between Bruch´s membrane, the retinal pigment epithelium and the photoreceptors as basis for a stable visual function. The concept of a sliding tissue layer has also been suggested previously by Chui and associates, who discussed that the existence of a difference between the photoreceptor margin and retinal pigment epithelium margin in some eyes may suggest that a tissue slippage occurs within the retina during eye growth [9]. The difference between both models is that Chui suggested a slippage within the retina, while in the present study, a slippage was postulated to occur between Bruch´s membrane and the sclera

The mean value of the fovea-disc border distance of 4.16 ± 0.44 mm as found in our study correlated with the findings obtained in previous investigations, if one takes into account that most of the previous studies measured the distance from the fovea to the center of the optic disc. In 51 preterm and full-term infants, mean optic disc-fovea distance was 4.4 ± 0.4 mm, without difference between the preterm infants and the full-term infants [38]. In 183 diabetic patients without retinopathy, van de Put and coworkers found a mean disc-fovea distance of 4.72 ± 0.27 mm, and a longer disc-fovea distance was associated with more myopic refractive error [25]. The disc-fovea distance in 27 prematurely born children at an age of 10–11 years was 4.74 ± 0.29 mm, as reported by Knaapi and coworkers [39]. In contrast to our study, Knaapi and associates did not find significant associations between the disc-fovea distance and refractive error (or axial length), although one child with axially high myopia had an above-average disc-fovea distance of 6.35 mm. Lee and colleagues measured the distance between the temporal optic disc margin to the fovea distance in 88 patients with normal-tension glaucoma [40]. They found that in patients with a sparing of the central field the disc margin-fovea distance (3.88 ± 0.28 mm) was longer (P = 0.002) than in the group with glaucomatous central visual field loss (3.64 ± 0.40 mm). Interestingly, parapapillary atrophy (beta zone) was wider (P = 0.03) in the group with sparing of the central visual field and longer disc margin-fovea distance. It fits with our observation, that a longer disc-fovea distance was significantly associated with larger parapapillary gamma zone.

We had excluded eyes with secondary macular Bruch´s membrane defects in our study, since the irregular arrangement of the parts of Bruch´s membrane made it impossible to reliably measure the length of Bruch´s membrane. Future studies may address whether in these eyes the distance between the optic disc and the foveal region is enlarged, then pointing to a potential association between this type of secondary Bruch´s membrane defects and scleral staphylomata at the posterior pole.

Our results should be interpreted with some limitations in mind. First, our study had a lower age limit of 50 years so that the findings of our study cannot directly be transferred on younger individuals. Second, our study included only a randomly selected group of individuals within each subgroup of axial length. The mean values of the MacBMLength and the width of parapapillary gamma zone are therefore not normative data of a population-based study population. Third, we assumed that the length of macular Bruch's membrane (“MacBMLength”), measured as distance between the fovea and the end of Bruch´s membrane in direction to the optic disc, was a surrogate for the photoreceptor density in the macular region. That assumption however, has not been proven. Fourth, the macula is a three-dimensional concave structure, centered on the fovea. In our analysis, only a single linear measurement from the fovea to the optic disc was taken into account. The interpretation to commenting on macular photoreceptor density has therefore to be taken with caution, as our study did not look at changes temporal, superior, and inferior to fovea center. In particular in eyes with posterior staphyloma, this point may be relevant. Fifth, the present study included Chinese individuals. Since ocular dimensions may differ between ethnicities, the measurements obtained in our study population may not directly be transferred onto other populations. Sixth, it was assumed that the length of the macular Bruch's membrane was a surrogate for the density of retinal pigment epithelium cells and macular photoreceptors. That assumption however, may be too simplistic, since only the inner part of Bruch´s membrane is produced by the retinal pigment epithelium.

In conclusion, the length of the macular Bruch´s membrane was not significantly associated with axial length. In contrast, larger width of parapapillary gamma and longer total fovea-disc border distance were strongly correlated with longer axial length. It suggests that the axial elongation associated increase in the fovea-disc distance predominantly occurs through a development or elongation of parapapillary gamma zone, while the macular Bruch´s membrane is mostly independent of axial elongation. One may infer that in axial elongation with a mostly constant macular Bruch´s membrane length, the density of retinal photoreceptors in the fovea remains unchanged.
